# Supplementary material for: In situ cryo-electron tomography reveals the progressive biogenesis of basal bodies and cilia in mouse ependymal cells
Source: Nat Commun. 2025 Jul 1;16:5932. doi: 10.1038/s41467-025-61015-6 (PMC12218126; doi:10.1038/s41467-025-61015-6)
Supplement: Supplementary file 1 — Supplementary Information [file 41467_2025_61015_MOESM1_ESM.pdf]

In situ cryo-electron tomography reveals the progressive biogenesis of basal bodies and cilia in mouse ependymal cells

Shanshan Ma[1,*], Luan Li[2,3,*], Zhixun Li[1,*], Shenjia Luo[1,*], Qi Liu[4,*], Wenjing Du[1], Benhua Qiu[2,3], Miao Gui[4,#], Xueliang Zhu[2,3,5,#], Qiang Guo[1,6,#]

[1] State Key Laboratory of Membrane Biology, Center for Life Sciences, Academy for Advanced Interdisciplinary Studies, School of Life Sciences, Peking University, Beijing 100871, China.

[2]Key Laboratory of Multi-Cell Systems, Shanghai Institute of Biochemistry and Cell Biology, Center for Excellence in Molecular Cell Science, Chinese Academy of Sciences, Shanghai 200031, China.

[3]University of Chinese Academy of Sciences, Beijing 100049, China.

[4]Department of Obstetrics and Gynecology, Sir Run Run Shaw Hospital, School of Medicine and Liangzhu Laboratory, Zhejiang University, Hangzhou 310058, China.

[5]Key Laboratory of Systems Health Science of Zhejiang Province, School of Life Science, Hangzhou Institute for Advanced Study, University of Chinese Academy of Sciences, Hangzhou 310024, China.

[6]Changping Laboratory, Beijing 102206, China.

[*]These authors contributed equally: Shanshan Ma, Luan Li, Zhixun Li, Shenjia Luo, Qi Liu.

[#]Correspondence: miaogui@zju.edu.cn (M.G.), xlzhu@sibcb.ac.cn (X.Z.), guo.qiang@pku.edu.cn (Q.G.)

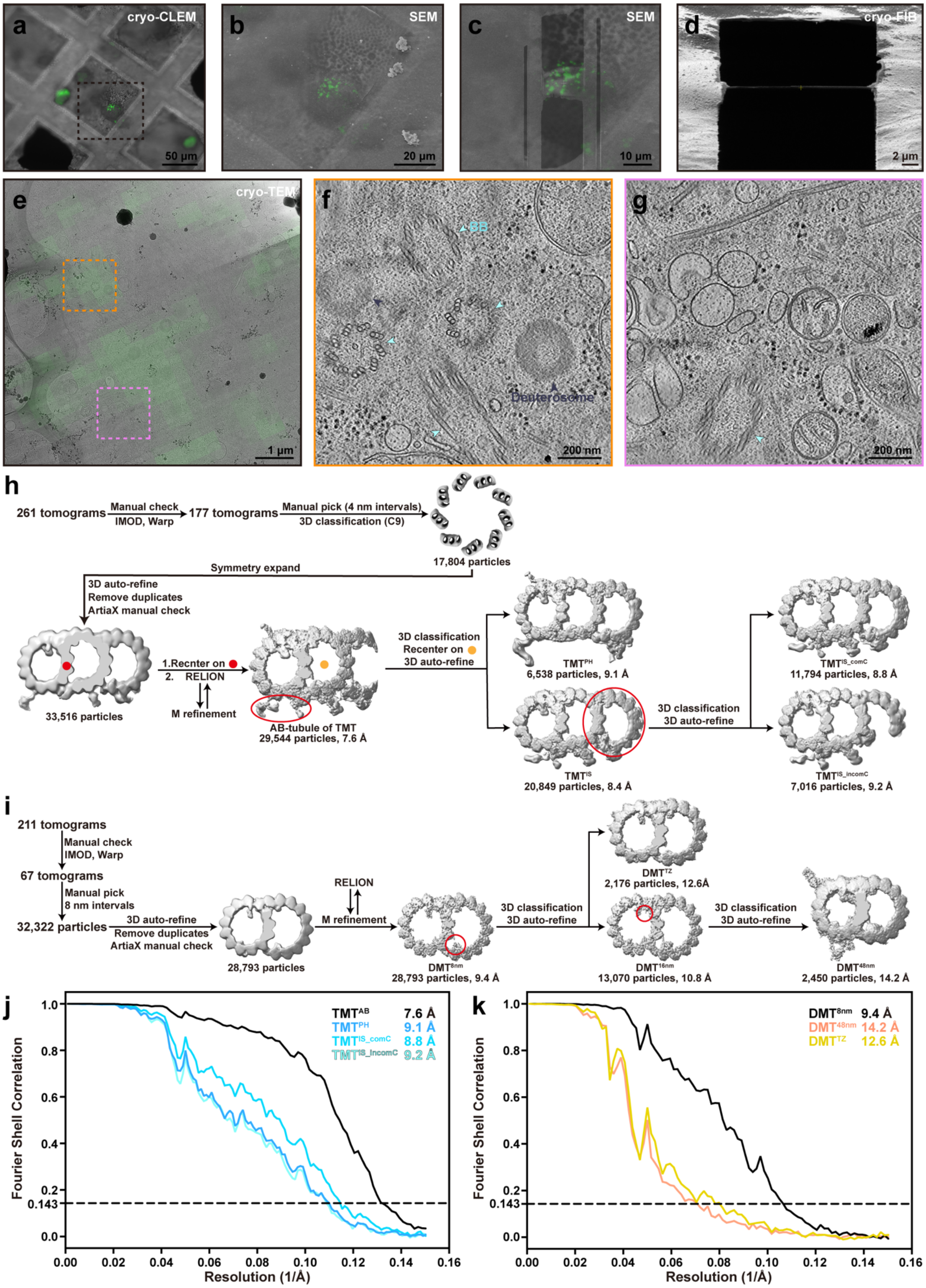

Suppementary Fig. 1. Workflow of sample preparation and data processing for cryo-ET.

(a) The cryo-fluorescence microscopy of mEPCs expressing GFP-Deup1. The dashed box

denotes the region of interest for cryo-FIB.

(b) The alignment and superimposition between the GFP image in (**a**) and corresponding SEM image of the target region.

(c) The final SEM image after lamella fabrication was aligned and overlayed with the GFP image in (**a**).

(**d**) The final FIB image of the lamella in (**c**) after milling.

(**e**) The low-dose cryo-TEM image of the lamella in (**c**) was aligned and superimposed with the GFP signal to show the target region for tilt-series acquisition.

(**f,g**) 2.7-nm-thick tomographic slices of the tomograms recorded in the orange box (**f**) and violet box (**g**) in (**e**). The BBs and deuterosomes are indicated by light blue arrowheads and dark blue arrowheads, respectively. BB, basal body.

(**h**) Schematic diagram of the data processing workflow to determine TMT structures. Basal bodies were manually picked and oversampled at 4 nm intervals. Symmetry expansion on the average map of basal body yielded 33,516 particles after 3D refinement to remove duplicates and discard poorly defined particles. A well-defined subset of 29,544 particles was selected and subjected to further refinement in RELION and M. Three classes (TMT^{PH}, TMT^{IS_comC} and TMT^{IS_incomC}) of TMT structures were determined by further 3D refinement, shifting particle coordinates and focused classification. Masks for classification are indicated by red circles.

(**i**) Schematic diagram of the data processing workflow to determine DMT structures. The axonemal microtubules were manually picked and sampled at 8 nm intervals. 28,793 DMT particles were yielded after 3D refinement to remove duplicates and poorly defined particles, which were subjected to further refinement in RELION and M. Focused classification revealed the 8-nm repeat DMT^{TZ} structure in transition zone and 48-nm repeat DMT^{48nm} structure in the axoneme beyond transition zone. Masks for classification are indicated by red circles.

(**j,k**) ‘Gold-standard’ Fourier shell correlation (FSC) curves of TMT (**j**) and DMT (**k**) structures.

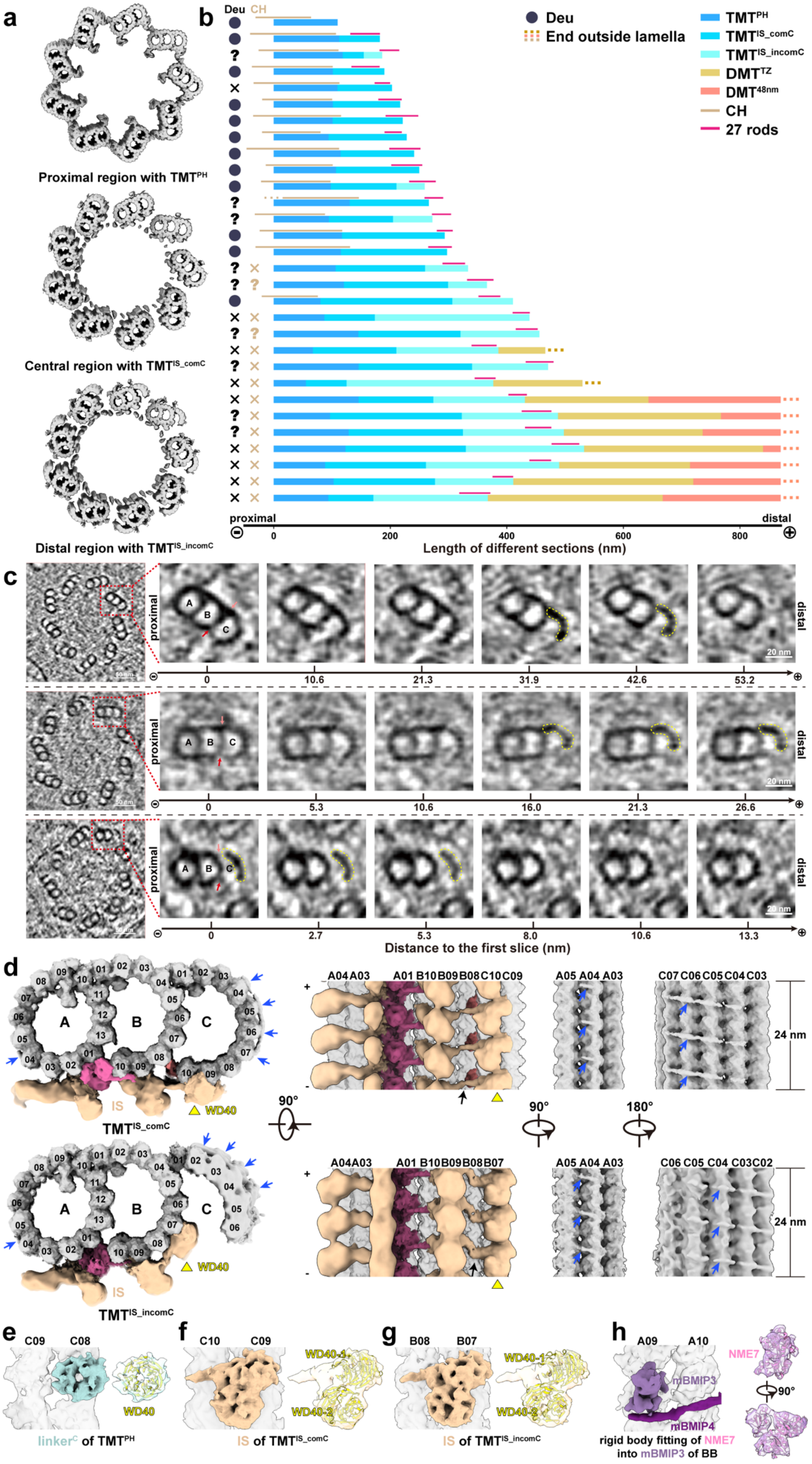
a
Proximal region with TMT^PH
Central region with TMT^IS_comC
Distal region with TMT^IS_incomC
b
Deu
CH
Deu
End outside lamella
TMT^PH
TMT^IS_comC
TMT^IS_incomC
DMT^TZ
DMT^48nm
CH
27 rods
proximal
distal
0
200
400
600
800
Length of different sections (nm)
c
proximal
distal
A
B
C
20 nm
0
10.6
21.3
31.9
42.6
53.2
5.3
16.0
26.6
2.7
8.0
13.3
Distance to the first slice (nm)
d
TMT^IS_comC
TMT^IS_incomC
IS
WD40
A04 A03
A01 B10 B09 B08 C10 C09
A05 A04 A03
C07 C06 C05 C04 C03
24 nm
90°
180°
A04 A03
A01 B10 B09 B08 B07
C06 C05 C04 C03 C02
e
C09
C08
WD40
linker^C of TMT^PH
f
C10
C09
WD40-1
WD40-2
IS of TMT^IS_comC
g
B08
B07
IS of TMT^IS_incomC
h
A09
A10
mBMIP3
mBMIP4
NME7
rigid body fitting of NME7 into mBMIP3 of BB

Suppementary Fig. 2. Distinct structural features of proximal, central and distal regions within basal bodies.

(a) Three cross sections with C9 symmetry represent the proximal region with TMT^{PH} (top), central region with $\text{TMT}^{\text{IS\_comC}}$ (middle) and distal region with $\text{TMT}^{\text{IS\_incomC}}$ (bottom) of BBs.

(b) Length measurement of the BBs and axonemes of mEPCs. The division of different sections was based on the results of 3D classification and the lengths were manually measured in tomograms. DMT^{48nm} represents the region of axonemes beyond TZ where DMT exhibits a 48-nm periodicity. 30 cilia with the definitive proximal end were determined and the distal end outside the lamella was denoted by '⋯' on the right end. The bottom axis represents the distance from the minus end of TMT, defined as the '0' origin point. Brown and red bars annotate the positions and lengths of the CH and 27 rods within BBs, respectively. The status of deuterosome and CH for each BB was marked individually on the left. Circles indicate the observed deuterosomes. 'X' marks suggest the absence of deuterosome and CH, while question marks denote the uncertainty due to being cropped by FIB milling. Deu, deuterosome; CH, central hub.

(c) 2.66-nm-thick serial slices from three TMTs revealing the progressive loss of protofilaments from proximal to distal BBs. Red and salmon arrows indicate the C10 and C01, respectively. Dashed yellow circles highlight the prolonged persistence of the incomplete C tubule.

(d) The structures of $\text{TMT}^{\text{IS\_comC}}$ and $\text{TMT}^{\text{IS\_incomC}}$ with higher counter levels compared to those in (**Fig. 2a-c**) show the distinct structural features. Yellow triangles denote the WD40 domains. Blue arrows indicate the striations outside A and C tubules. Black arrows annotate the connection between WD40 domain-containing proteins and the protrusions from B09. IS, inner scaffold.

(e-g) The magnified views of WD40 domain-containing proteins in the A-C linker of TMT^{PH} (**e**), IS of $\text{TMT}^{\text{IS\_comC}}$ (**f**) and $\text{TMT}^{\text{IS\_incomC}}$ (**g**). WD40 domains were fitted into the corresponding densities (right).

(h) The magnified view of mBMIP3 within BBs (left) and rigid-body fitting of NME7 from bovine respiratory cilia (PDB: 7RRO) into the mBMIP3 density map (right).

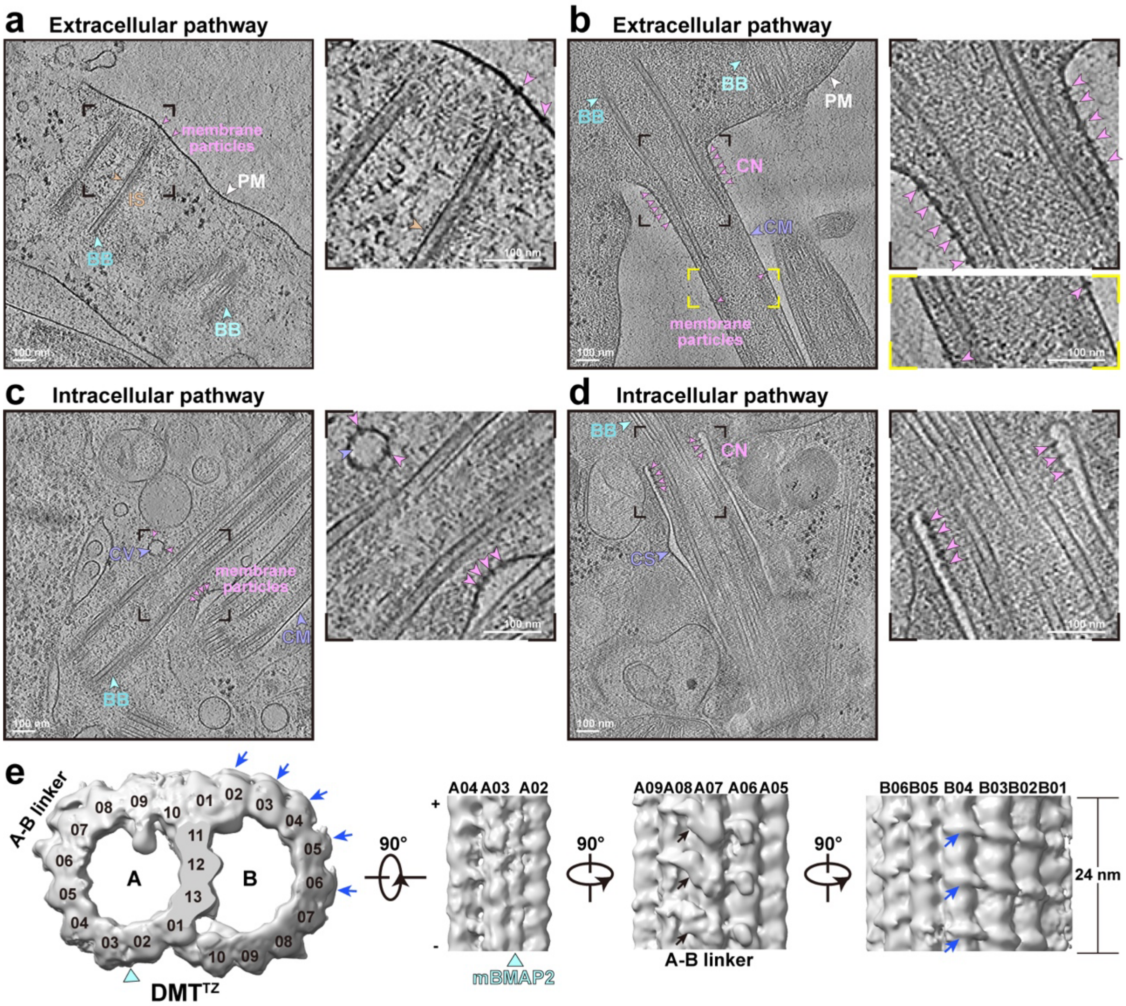

Suppementary Fig. 3. Basal bodies template ciliogenesis via both extracellular and intracellular pathways.

(a,b) 2.7-nm-thick tomographic slices (left) reveal the extracellular pathway that basal bodies migrate and dock to the plasma membrane (**a**), which results in the surfaced cilia formation (**b**). Enlarged views of the 16-nm-thick tomographic slices (right) show the distal basal body (**a**) and transition zone (**b**). The yellow box highlights the bead-like particles similar with that of ciliary necklace on the ciliary membrane beyond transition zone.

(c,d) 2.7-nm-thick tomographic slices (left) reveal the intracellular pathway by the observations of ciliary vesicles around bare axonemal microtubules (**c**), as well as the submerged cilia with ciliary sheath in the cytoplasm (**d**). Enlarged views of the 16-nm-thick tomographic slices (right) show the corresponding transition zone. BB, basal body; IS, inner scaffold; PM, plasma membrane; CM, ciliary membrane; CV, ciliary vesicle; CN, ciliary necklace, CS, ciliary sheath.

(e) The structural analysis of the 8-nm repeat DMT^{TZ} with a higher counter level compared to that in (**Fig. 3c**) highlights mBMAP2, A-B linker and the striation (blue arrows) traversing B tubule.

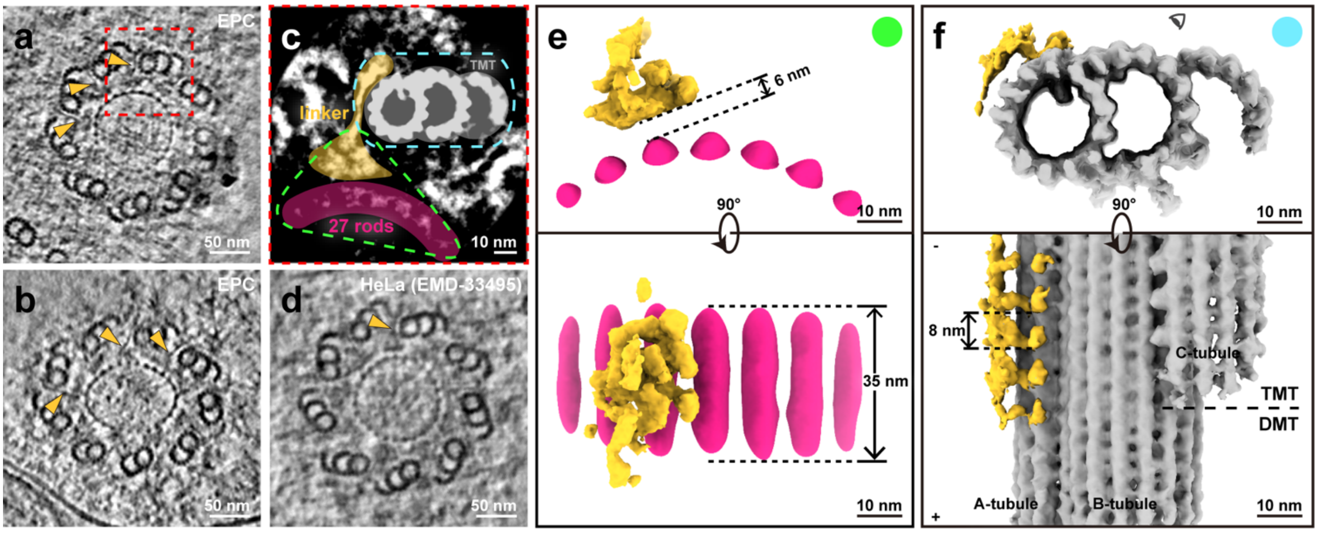

Suppementary Fig. 4. The junctions between basal bodies and 27 rods.

(a,b) 20-nm-thick tomographic slices show the T-shaped density (yellow arrowheads) linking the 27 rods with A tubule of basal bodies in mEPCs. The regions indicated in red box from our data were extracted for the averaging of the 27 rods.

(c) The 2D projection of the averaged structure in (**a**) within the red box represents the TMT, 27 rods and connecting T-shaped density. The areas shown in green and blue dotted lines were masked respectively for averaging.

(d) Similar structures in (**c**) were also observed in the centriole of HeLa cells (EMD-33495). Tomographic thickness, 20 nm.

(e) The averaged structure of 27 rods and the head of T-shaped density indicated in (**c**) within the green dotted line.

(f) The averaged structure of TMT and the stalk of T-shaped density indicated in (**c**) within the blue dotted line.

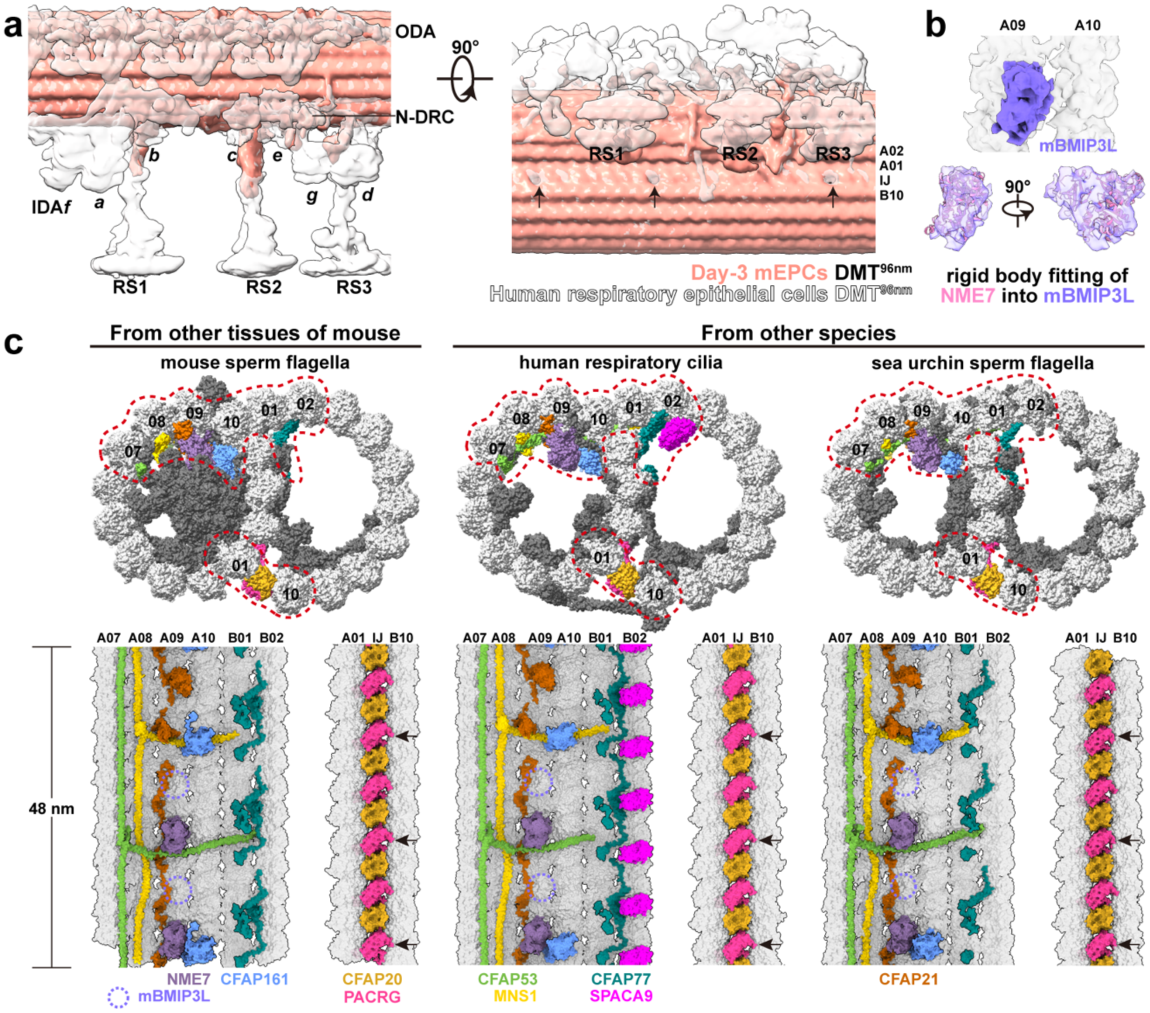

Suppementary Fig. 5. The absence of PACRG and redundancy of MIPs in nascent cilia from day-3 mEPCs.

(a) Structural comparison of the 96-nm repeat DMT structures in cilia from day-3 mEPCs and human respiratory epithelial cells (EMD-35888). Black arrows annotate the sites of PACRG absence in day-3 mEPCs. RS, radial spokes; ODA, outer dynein arms; IDA, inner dynein arms; N-DRC, nexin-dynein regulatory complexes.

(b) The magnified view of mBMIP3L within nascent cilia from day-3 mEPCs (top) and rigid-body fitting of NME7 from bovine respiratory cilia (PDB: 7RRO) into the mBMIP3L density map (bottom).

(c) Structural analysis of mature DMTs in mouse sperm flagella (PDB: 8IYJ), human respiratory cilia (PDB: 8J07) and sea urchin sperm flagella (PDB: 8SNB). MIPs within the region outlined by the red dashed lines were colored. The cross sections (top) and the longitudinal sections (bottom) depict the shedding of mBMIP5 and mBMIP3L, as well as

PACRG with 8-nm repeat in mature cilia/flagella. Black arrows annotate the sites where PACRG is absent in nascent cilia from day-3 mEPCs. Purple dashed circles denote the absence of mBMIP3L in mature cilia/flagella. CFAP77, rather than mBMIP5, binds to B01 and B02 in mature cilia/flagella. IJ, inner junction.

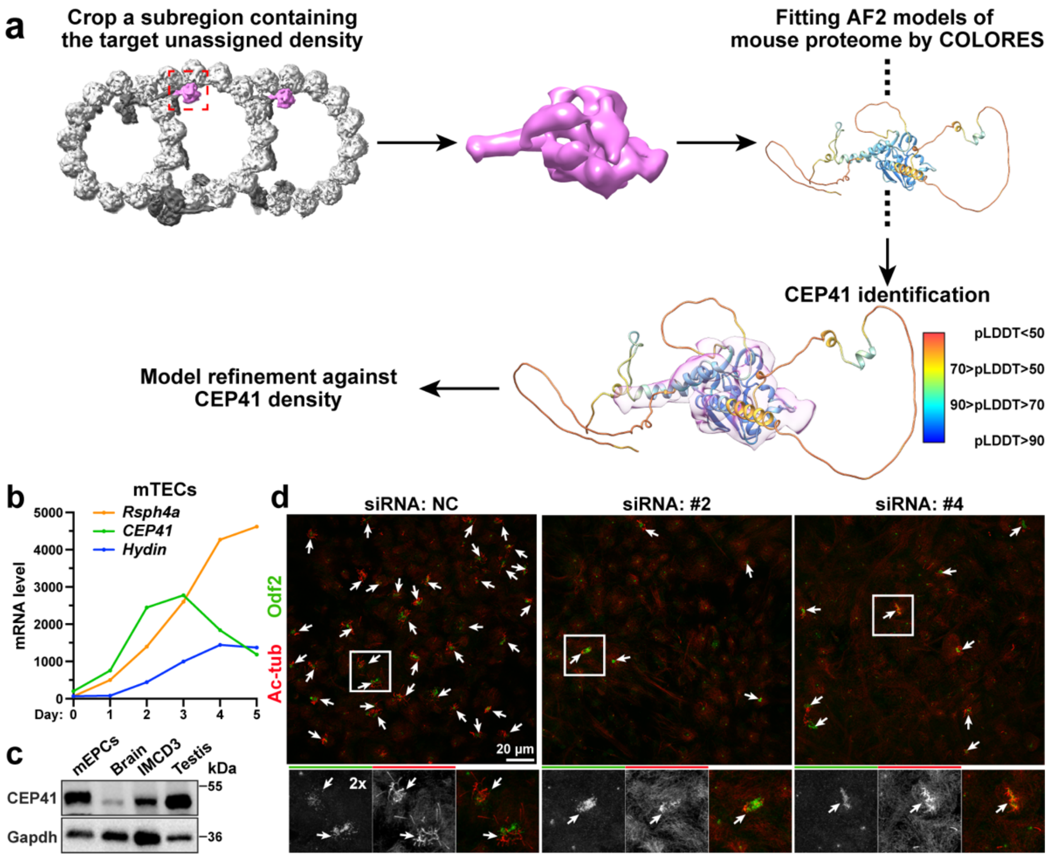

Suppementary Fig. 6. Identification of CEP41 and its role in multiciliogenesis.

(a) A flow diagram showing how CEP41 was identified based on AlphaFold2 model fitting. The unassigned density in TMT was cropped and AlphaFold2 models of mouse proteome were systematically fitted into the density using COLORES program in Situs[1-3]. CEP41 was identified based on the fitting scores and manual inspection. The core of CEP41 AlphaFold2 model with high predicted local-distance difference test (pLDDT) values fits well with the cryo-EM density while the flexible regions with low pLDDT values stretch out of the cryo-EM density, suggesting a good fitting. The CEP41 AlphaFold2 model was then manually adjusted and refined against the cryo-EM density.

(b) *CEP41* was upregulated during multiciliated cell differentiation. Expression profiles of *Rsph4a* and *Hydin*, genes respectively encoding a radial spoke component and CP component[4, 5], were used to reflect the progression of multiciliogenesis. The data were from cDNA microarray results on mTECs[6] cultured at an air-liquid interface for the indicated days to induce multiciliation.

(c) CEP41 was highly expressed in cells abundant in motile cilia. Day-10 mEPCs were used for immunoblotting, whereas IMCD3 cells were cultured to day 2 post serum starvation to

induce primary cilia[7]. Brain and testis tissues were dissected from a 2-month-old male mouse. Gapdh served as a loading control.

(d) Depletion of CEP41 inhibited multicilia formation. mEPCs treated as in (**Fig. 6g,h**) were immunostained for acetylated tubulin (Ac-tub) and Odf2, a distal basal body protein[8], to visualize cilia and basal bodies, respectively. Multiciliated cells are indicated by arrows. Framed regions were magnified to show details. NC, negative control. Source data are provided as a Source Data file.

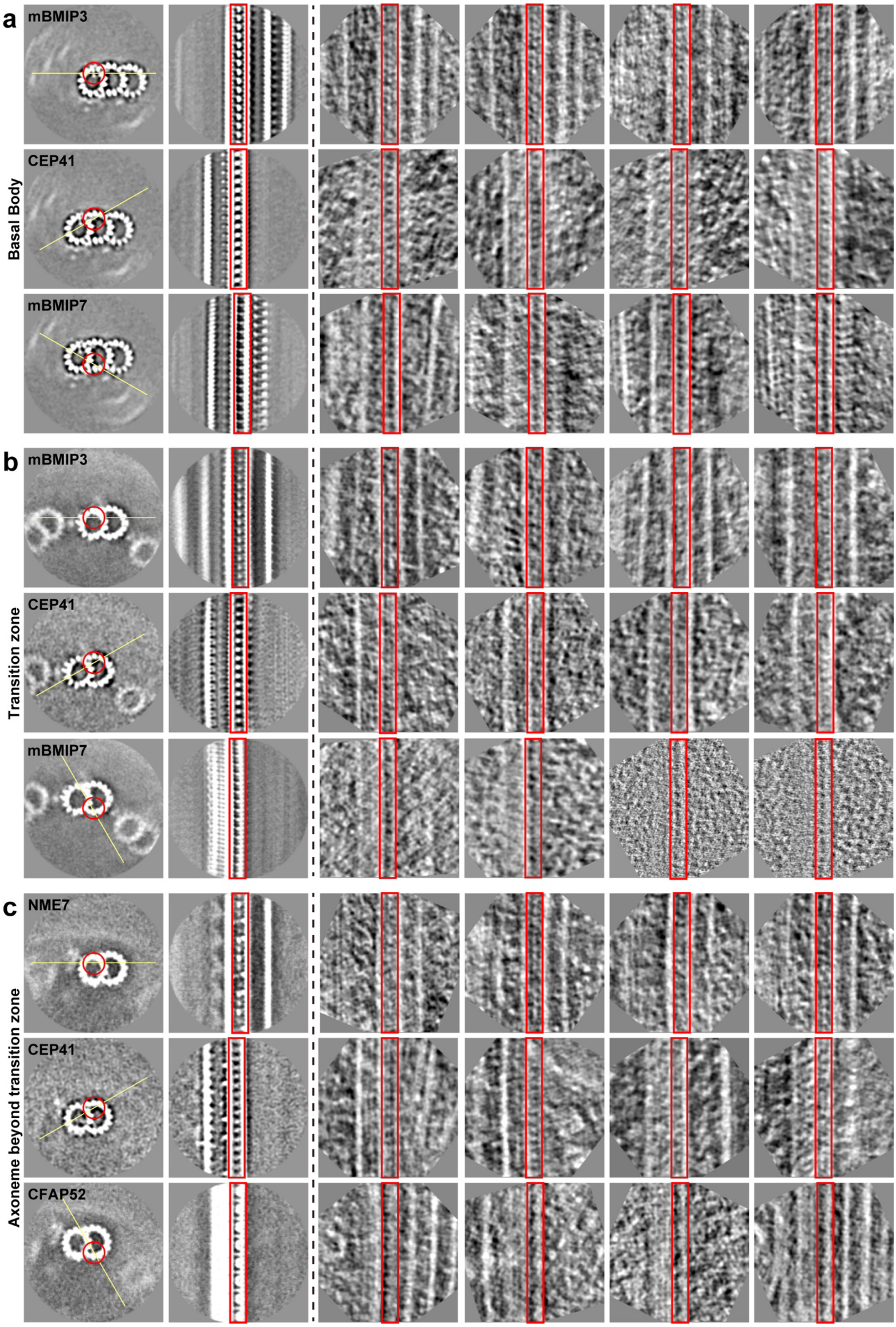
a
mBMIP3
CEP41
Basal Body
mBMIP7
b
mBMIP3
CEP41
Transition zone
mBMIP7
c
NME7
CEP41
Axoneme beyond transition zone
CFAP52

Suppementary Fig. 7. 2D projection of MIPs reveals their periodicity.

(a) The 2D projections of mBMIP3, CEP41 and mBMIP7 in TMT. Left of dotted line: the 2D projection of averaged structure from cross-section view and clip view (along the yellow line); Right of dotted line: the 2D projection of four cropped particles rotated to the same direction of clip view. The target MIPs were marked in red circles or rectangular boxes.

(b,c) The 2D projections of the MIPs in the same position of (**a**) but in transition zone (**b**), as well as in nascent axonemes beyond transition zone (**c**).

Supplementary Table 1. Cryo-EM data collection, refinement and validation statistics.

	TMT^{AB}	TMT^{IS_comC}	TMT^{PH}	TMT^{IS_incomC}	DMT^{TZ}	DMT^{48nm}	CEP41
**Sample**				Day-3 mEPCs			
**Data collection and processing**							
Image detector	GATAN K3	GATAN K3	GATAN K3	GATAN K3	GATAN K3	GATAN K3	GATAN K3
Magnification	26,000×	26,000×	26,000×	26,000×	26,000×	26,000×	26,000×
Voltage (kV)	300	300	300	300	300	300	300
Total electron exposure (e^{-}/Å^2)	110	110	110	110	110	110	110
Defocus range (μm)	-3.0 to -5.0	-3.0 to -5.0	-3.0 to -5.0	-3.0 to -5.0	-3.0 to -5.0	-3.0 to -5.0	-3.0 to -5.0
Tilt angle range(°)	+66 to -38	+66 to -38	+66 to -38	+66 to -38	+66 to -38	+66 to -38	+66 to -38
Pretilt (°)	+14	+14	+14	+14	+14	+14	+14
Tilt step angle(°)	2	2	2	2	2	2	2
Pixel size (Å)	3.328	3.328	3.328	3.328	3.328	3.328	3.328
Symmetry imposed	C1	C1	C1	C1	C1	C1	C1
Applied periodicity (nm)	8	8	8	8	8	48	8
Initial subtomograms	33,516	33,516	33,516	33,516	32,322	32,322	47,876
Final subtomograms	29,544	11,794	6,538	7,016	2,176	2,450	44,672
Map resolution (Å)	7.6	8.8	9.1	9.2	12.6	14.2	8.1
FSC threshold	0.143	0.143	0.143	0.143	0.143	0.143	0.143
EMDB code	EMD-39652	EMD-39653	EMD-39654	EMD-39655	EMD-39656	EMD-39657	EMD-39658

References

1. Jumper J, *et al.* Highly accurate protein structure prediction with AlphaFold. *Nature* **596**, 583-589 (2021).
2. Wriggers W. Conventions and workflows for using Situs. *Acta Crystallogr D Biol Crystallogr* **68**, 344-351 (2012).
3. Wriggers W, Milligan RA, McCammon JA. Situs: A package for docking crystal structures into low-resolution maps from electron microscopy. *Journal of structural biology* **125**, 185-195 (1999).
4. Zheng W, *et al.* Distinct architecture and composition of mouse axonemal radial spoke head revealed by cryo-EM. *Proceedings of the National Academy of Sciences of the United States of America* **118**, e2021180118 (2021).
5. Lechtreck KF, Delmotte P, Robinson ML, Sanderson MJ, Witman GB. Mutations in Hydin impair ciliary motility in mice. *J Cell Biol* **180**, 633-643 (2008).
6. Xu Y, *et al.* Characterization of tetratricopeptide repeat-containing proteins critical for cilia formation and function. *PloS one* **10**, e0124378 (2015).
7. Cao J, *et al.* miR-129-3p controls cilia assembly by regulating CP110 and actin dynamics. *Nat Cell Biol* **14**, 697-706 (2012).
8. Ishikawa H, Kubo A, Tsukita S, Tsukita S. Odf2-deficient mother centrioles lack distal/subdistal appendages and the ability to generate primary cilia. *Nat Cell Biol* **7**, 517-524 (2005).
