# Supplementary material for: In situ cryo-electron tomography reveals the progressive biogenesis of basal bodies and cilia in mouse ependymal cells
Source: Nat Commun. 2025 Jul 1;16:5932. doi: 10.1038/s41467-025-61015-6 (PMC12218126; doi:10.1038/s41467-025-61015-6)
Supplement: Supplementary file 2 — Description of Additional Supplementary Files [file 41467_2025_61015_MOESM2_ESM.pdf]

Description of Additional Supplementary Files

Supplementary Movies

Supplementary Movie 1. Deuterosome-dependent basal body biogenesis at stage-III in mEPCs.

Supplementary Movie 2. Motilities of day-3 short cilia. Multicilia in living mEPCs were stained with SiR-tubulin, followed by high-speed imaging at 60-ms intervals.

Supplementary Movie 3. Motilities of day-3 medium cilia. Multicilia in living mEPCs were stained with SiR-tubulin, followed by high-speed imaging at 60-ms intervals.

Supplementary Movie 4. Motilities of day-3 long cilia. Multicilia in living mEPCs were stained with SiR-tubulin, followed by high-speed imaging at 60-ms intervals.

Supplementary Movie 5. Motilities of day-10 cilia. Multicilia in living mEPCs were stained with SiR-tubulin, followed by high-speed imaging at 50-ms intervals.

Supplementary Movie 6. Fitting of CEP41 into the density of mBMIP5. Residues outside the density were omitted for clarity.
