# Supplementary material for: In situ cryo-electron tomography reveals the progressive biogenesis of basal bodies and cilia in mouse ependymal cells
Source: Nat Commun. 2025 Jul 1;16:5932. doi: 10.1038/s41467-025-61015-6 (PMC12218126; doi:10.1038/s41467-025-61015-6)
Supplement: Supplementary file 9 — Reporting Summary [file 41467_2025_61015_MOESM9_ESM.pdf]

nature portfolio

Corresponding author(s): Qiang Guo

Last updated by author(s): May 21, 2025

Reporting Summary

Nature Portfolio wishes to improve the reproducibility of the work that we publish. This form provides structure for consistency and transparency in reporting. For further information on Nature Portfolio policies, see our Editorial Policies and the Editorial Policy Checklist.

Statistics

For all statistical analyses, confirm that the following items are present in the figure legend, table legend, main text, or Methods section.

n/a	Confirmed	
☐	☒	The exact sample size (n) for each experimental group/condition, given as a discrete number and unit of measurement
☐	☒	A statement on whether measurements were taken from distinct samples or whether the same sample was measured repeatedly
☐	☒	The statistical test(s) used AND whether they are one- or two-sided<br>*Only common tests should be described solely by name; describe more complex techniques in the Methods section.*
☐	☒	A description of all covariates tested
☐	☒	A description of any assumptions or corrections, such as tests of normality and adjustment for multiple comparisons
☐	☒	A full description of the statistical parameters including central tendency (e.g. means) or other basic estimates (e.g. regression coefficient) AND variation (e.g. standard deviation) or associated estimates of uncertainty (e.g. confidence intervals)
☐	☒	For null hypothesis testing, the test statistic (e.g. F, t, r) with confidence intervals, effect sizes, degrees of freedom and P value noted<br>*Give P values as exact values whenever suitable.*
☒	☐	For Bayesian analysis, information on the choice of priors and Markov chain Monte Carlo settings
☒	☐	For hierarchical and complex designs, identification of the appropriate level for tests and full reporting of outcomes
☒	☐	Estimates of effect sizes (e.g. Cohen's d, Pearson's r), indicating how they were calculated

Our web collection on statistics for biologists contains articles on many of the points above.

Software and code

Policy information about availability of computer code

Data collection	SerialEM v4.1; PACE-tomo (https://github.com/eisfabian/PACEtomo)
Data analysis	Maps v3.13.10; TOMOMAN (https://github.com/wan-lab-vanderbilt/TOMOMAN/tree/08042020); MATLAB R2019b; MotionCor2 v1.5.0; IMOD v4.11.3; IsoNet 0.2; MemBrain-Seg (https://github.com/teamtomo/membrain-seg); Amira 2020.3; ChimeraX v1.6.1; RELION-2.1; Coot v0.9.4.1; Situs v3.2; Cellsens v4.2; ImageJ v1.53r; GraphPad Prism v9.0.0

For manuscripts utilizing custom algorithms or software that are central to the research but not yet described in published literature, software must be made available to editors and reviewers. We strongly encourage code deposition in a community repository (e.g. GitHub). See the Nature Portfolio guidelines for submitting code & software for further information.

Data

Policy information about availability of data

All manuscripts must include a data availability statement. This statement should provide the following information, where applicable:

- Accession codes, unique identifiers, or web links for publicly available datasets
- A description of any restrictions on data availability
- For clinical datasets or third party data, please ensure that the statement adheres to our policy

The subtomogram averaging maps from mEPCs in the study have been deposited in the Electron Microscopy Data Bank (EMDB) with the following accession numbers: EMD-39652 (8-nm repeat TMTAB), EMD-39654 (8-nm repeat TMTPH), EMD-39653 (8-nm repeat TMTIS_comC), EMD-39655 (8-nm repeat

TMTIS_incomC), EMD-39656 (8-nm repeat DMTTZ), EMD-39657 (48-nm repeat DMT48nm), EMD-39658 (8-nm repeat CEP41). The tomograms analyzed in Fig. 1 have been deposited at the EMDB with accession numbers: EMD-39659 (Fig. 1g) and EMD-39660 (Fig. 1e and Supplementary Video 1). To achieve broader accessibility, more tomograms depicting the deuterosome-dependent basal body biogenesis at various stages have been deposited at the EMDB with accession numbers: EMD-63282, EMD-63283, EMD-63284, EMD-63285, EMD-63286, EMD-63287, EMD-63288 and EMD-63289.

Research involving human participants, their data, or biological material

Policy information about studies with human participants or human data. See also policy information about sex, gender (identity/presentation), and sexual orientation and race, ethnicity and racism.

Reporting on sex and gender	*Use the terms sex (biological attribute) and gender (shaped by social and cultural circumstances) carefully in order to avoid confusing both terms. Indicate if findings apply to only one sex or gender; describe whether sex and gender were considered in study design; whether sex and/or gender was determined based on self-reporting or assigned and methods used. Provide in the source data disaggregated sex and gender data, where this information has been collected, and if consent has been obtained for sharing of individual-level data; provide overall numbers in this Reporting Summary. Please state if this information has not been collected. Report sex- and gender-based analyses where performed, justify reasons for lack of sex- and gender-based analysis.*
Reporting on race, ethnicity, or other socially relevant groupings	*Please specify the socially constructed or socially relevant categorization variable(s) used in your manuscript and explain why they were used. Please note that such variables should not be used as proxies for other socially constructed/relevant variables (for example, race or ethnicity should not be used as a proxy for socioeconomic status). Provide clear definitions of the relevant terms used, how they were provided (by the participants/respondents, the researchers, or third parties), and the method(s) used to classify people into the different categories (e.g. self-report, census or administrative data, social media data, etc.) Please provide details about how you controlled for confounding variables in your analyses.*
Population characteristics	*Describe the covariate-relevant population characteristics of the human research participants (e.g. age, genotypic information, past and current diagnosis and treatment categories). If you filled out the behavioural & social sciences study design questions and have nothing to add here, write "See above."*
Recruitment	*Describe how participants were recruited. Outline any potential self-selection bias or other biases that may be present and how these are likely to impact results.*
Ethics oversight	*Identify the organization(s) that approved the study protocol.*

Note that full information on the approval of the study protocol must also be provided in the manuscript.

Field-specific reporting

Please select the one below that is the best fit for your research. If you are not sure, read the appropriate sections before making your selection.

☒ Life sciences ☐ Behavioural & social sciences ☐ Ecological, evolutionary & environmental sciences

For a reference copy of the document with all sections, see nature.com/documents/nr-reporting-summary-flat.pdf

Life sciences study design

All studies must disclose on these points even when the disclosure is negative.

Sample size	Sample sizes were not predetermined with any statistic method and it is chosen based on availability and general guideline to ensure at least two independent experiments with sufficient reproducibility. All samples sizes are included in the figure legends and Methods.
Data exclusions	1. For cryo-ET data processing, images with weak contrast or ice contamination at high tilt angles were discarded. Particles were excluded during sub-tomogram averaging when their coordinates were in the wrong periodicity.<br>2. For other analysis, no data were excluded.
Replication	Reproducibility was confirmed and the experimental replicates are stated in the figure legends and Methods.
Randomization	1. For calculation of the Fourier Shell Correlation (FSC), subtomograms were randomly split into two halves using the RELION software.<br>2. For other analysis, samples were allocated randomly for culture and analysis. Cells from mulitiple randomly-chosen fields were used in each experiment.
Blinding	N/A

Reporting for specific materials, systems and methods

We require information from authors about some types of materials, experimental systems and methods used in many studies. Here, indicate whether each material, system or method listed is relevant to your study. If you are not sure if a list item applies to your research, read the appropriate section before selecting a response.

Materials & experimental systems

n/a	Involved in the study
☐	☒ Antibodies
☐	☒ Eukaryotic cell lines
☒	☐ Palaeontology and archaeology
☐	☒ Animals and other organisms
☒	☐ Clinical data
☒	☐ Dual use research of concern
☒	☐ Plants

Methods

n/a	Involved in the study
☒	☐ ChIP-seq
☒	☐ Flow cytometry
☒	☐ MRI-based neuroimaging

Antibodies

Antibodies used

The information of all the antibodies used in the study are presented in the order of supplier name, catalog name, clone name, lot number and dilution.
For primary antibodies,
Anti-CEP41: Affinity Biosciences, DF9362, rabbit, 89H4950, 1:2000 dilution.
Anti-Gapdh: Proteintech, 10494-1-AP, rabbit, 00103483, 1:5000 dilution.
Anti-acetylated-tubulin: Sigma, T6793(6-11B-1), mouse, 0000269851, 1:1000 dilution.
Anti-Odf2: home-made, NA, guinea pig, NA, 1:500 dilution.
For secondary antibodies,
HRP-conjugated goat anti-Rabbit IgG (H+L): Thermo Fisher Scientific, G-21234, goat, 2156243, 1:5000 dilution.
Alexa Fluor 647-conjugated goat anti-Rabbit IgG (H+L): Thermo Fisher Scientific, A-21245, goat, 2442141, 1:1000 dilution.
CyTM3-conjugated donkey anti-mouse IgG (H+L): Jackson ImmunoResearch, 715-165-151, donkey, 162530, 1:1000 dilution.
Alexa Fluor 488-conjugated donkey anti-guinea pig IgG (H+L): Jackson ImmunoResearch, 706-545-148, donkey, 158591, 1:1000 dilution.

Validation

All commercially available primary antibodies have been validated by the manufactures.
Acetylated-tubulin (Mouse): For IF, ciliated cells sample yielded the anticipated localization pattern, verifying antibody specificity in the lab. (https://www.sigmaaldrich.cn/CN/en/product/sigma/t6793?context=product)
CEP41 (Rabbit): Specificity for WB was confirmed by detecting the expected band in a rat liver or mouse brain preparation and absence in negative controls. Specificity for IF was confirmed by detecting the anticipated localization pattern in A549 cells sample. (https://www.affbiotech.com/goods-12835-DF9362-CEP41_Antibody.html)
Gapdh (Rabbit): Specificity for WB was confirmed by detecting the expected band in HeLa cells, HepG2 cells, ROS1728 cells, pig brain tissue, zebrafish tissue, whole yeast, whole Nematode tissue, soybean whole plant tissue, arabidopsis whole plant tissue, HEK-293 cells, Jurkat cells, K-562 cells, HSC-T6 cells, NIH/3T3 cells, 4T1 cells, C6 cells, PC-12 cells, C2C12 cells, SP2/0 cells, rat brain tissue, mouse brain tissue and absence in negative controls. (https://www.ptglab.com/products/GAPDH-Antibody-60004-1-Ig.htm)
Odf2 (Guinea Pig, Homemade): For IF, ciliated cells sample yielded the anticipated localization pattern, verifying antibody specificity in the lab (https://doi.org/10.1038/s41467-021-21506-8).

Eukaryotic cell lines

Policy information about cell lines and Sex and Gender in Research

Cell line source(s)

HEK293T (ATCC, catalogue no. CRL-3216), IMCD3 (ATCC, catalogue no. CRL-2123).

Authentication

The cell lines have been authenticated by the vendor. No further authentication was performed.

Mycoplasma contamination

The cell lines were tested negative for mycoplasma contamination.

Commonly misidentified lines
(See ICLAC register)

No commonly misidentified cell lines were used in the study .

Animals and other research organisms

Policy information about studies involving animals; ARRIVE guidelines recommended for reporting animal research, and Sex and Gender in Research

Laboratory animals

Wild-type P0 mice (C57BL/6J) were used for primary cell culture. Wild-type 2-month-old mice (C57BL/6J) were used dissect the brain and testis tissues. The mice were housed under specific-pathogen-free (SPF) conditions in cages and a 12/12-hr light/dark photoperiod at 20-26 °C. The humidity of the housing room was maintained at 40-70% humidity.

Wild animals

No wild animals were used in this study.

Reporting on sex

Both females and males were used to dissect and culture mEPCs. A male mouse was used to dissect the brain and testis tissues.

Field-collected samples

No filed-collected samples were used in this study.

Ethics oversight	Experiments involving mouse tissues were performed in accordance with protocols approved by the Institutional Animal Care and Use Committee of CAS Center for Excellence in Molecular Cell Science, Institute of Biochemistry and Cell Biology, Chinese Academy of Sciences.

Note that full information on the approval of the study protocol must also be provided in the manuscript.

Plants

Seed stocks	*Report on the source of all seed stocks or other plant material used. If applicable, state the seed stock centre and catalogue number. If plant specimens were collected from the field, describe the collection location, date and sampling procedures.*
Novel plant genotypes	*Describe the methods by which all novel plant genotypes were produced. This includes those generated by transgenic approaches, gene editing, chemical/radiation-based mutagenesis and hybridization. For transgenic lines, describe the transformation method, the number of independent lines analyzed and the generation upon which experiments were performed. For gene-edited lines, describe the editor used, the endogenous sequence targeted for editing, the targeting guide RNA sequence (if applicable) and how the editor was applied.*
Authentication	*Describe any authentication procedures for each seed stock used or novel genotype generated. Describe any experiments used to assess the effect of a mutation and, where applicable, how potential secondary effects (e.g. second site T-DNA insertions, mosiacism, off-target gene editing) were examined.*
